# Submicroscopic and asymptomatic *Plasmodium falciparum* and *Plasmodium vivax* infections are common in western Thailand - molecular and serological evidence

**DOI:** 10.1186/s12936-015-0611-9

**Published:** 2015-02-25

**Authors:** Elisabeth Baum, Jetsumon Sattabongkot, Jeeraphat Sirichaisinthop, Kirakorn Kiattibutr, D Huw Davies, Aarti Jain, Eugenia Lo, Ming-Chieh Lee, Arlo Z Randall, Douglas M Molina, Xiaowu Liang, Liwang Cui, Philip L Felgner, Guiyun Yan

**Affiliations:** Department of Medicine, Division of Infectious Diseases, University of California Irvine, Irvine, CA USA; Mahidol Vivax Research Unit, Faculty of Tropical Medicine, Mahidol University, Bangkok, Thailand; Vector-Borne Disease Training Center, Saraburi, Thailand; Program in Public Health, University of California Irvine, Irvine, CA USA; Antigen Discovery Inc., Irvine, CA USA; Department of Entomology, School of Agricultural Sciences, Pennsylvania State University, University Park, PA USA

**Keywords:** Asymptomatic, Submicroscopic, Plasmodium vivax, Plasmodium falciparum, Mixed-species, Thailand, Southeast Asia, Molecular screening, qPCR, Protein microarray, Antibodies, Serology, Surveillance

## Abstract

**Background:**

Malaria is a public health problem in parts of Thailand, where *Plasmodium falciparum* and *Plasmodium vivax* are the main causes of infection. In the northwestern border province of Tak parasite prevalence is now estimated to be less than 1% by microscopy. Nonetheless, microscopy is insensitive at low-level parasitaemia. The objective of this study was to assess the current epidemiology of falciparum and vivax malaria in Tak using molecular methods to detect exposure to and infection with parasites; in particular, the prevalence of asymptomatic infections and infections with submicroscopic parasite levels.

**Methods:**

Three-hundred microlitres of whole blood from finger-prick were collected into capillary tubes from residents of a sentinel village and from patients at a malaria clinic. Pelleted cellular fractions were screened by quantitative PCR to determine parasite prevalence, while plasma was probed on a protein microarray displaying hundreds of *P. falciparum* and *P. vivax* proteins to obtain antibody response profiles in those individuals.

**Results:**

Of 219 samples from the village, qPCR detected 25 (11.4%) *Plasmodium* sp. infections, of which 92% were asymptomatic and 100% were submicroscopic. Of 61 samples from the clinic patients, 27 (44.3%) were positive by qPCR, of which 25.9% had submicroscopic parasite levels. Cryptic mixed infections, misdiagnosed as single-species infections by microscopy, were found in 7 (25.9%) malaria patients. All sample donors, parasitaemic and non-parasitaemic alike, had serological evidence of parasite exposure, with 100% seropositivity to at least 54 antigens. Antigens significantly associated with asymptomatic infections were *P. falciparum* MSP2, DnaJ protein, putative E1E2 ATPase, and three others.

**Conclusion:**

These findings suggest that parasite prevalence is higher than currently estimated by local authorities based on the standard light microscopy. As transmission levels drop in Thailand, it may be necessary to employ higher throughput and sensitivity methods for parasite detection in the phase of malaria elimination.

**Electronic supplementary material:**

The online version of this article (doi:10.1186/s12936-015-0611-9) contains supplementary material, which is available to authorized users.

## Background

Malaria is a major public health problem in Southeast Asia, including parts of Thailand, where its epidemiology is complicated by great geographical heterogeneity in disease endemicity, the presence of five *Plasmodium* species that cause human disease (*Plasmodium falciparum*, *Plasmodium vivax, Plasmodium malariae*, *Plasmodium ovale and Plasmodium knowlesi* [1,2]) and diverse vector systems with different vectorial capacities for the parasites [3]. A major challenge for control and elimination of malaria in this region is accurate diagnosis, including parasite species identification, particularly of those infections in asymptomatic individuals who may act as silent reservoirs and maintain parasite transmission in their communities [4,5].

In Thailand, malaria control efforts have been highly effective in curbing the infection nationwide [6]. Nonetheless, malaria is still endemic along the hilly and forested areas of the country’s borders with Myanmar and Cambodia, where transmission levels vary widely [7-9]. The northwestern province of Tak, bordering with Myanmar, historically had the highest parasite prevalence in the country [8-10] and has been the focus of intense malaria control measures for decades [11]. As a result, in 2011–2013, parasite prevalence was found to be <1% in cross-sectional surveys of several sentinel villages (Thai Ministry of Public Health, Bureau of Vector-Borne Disease surveillance report, unpublished). In the same period, of the febrile individuals seeking treatment at local malaria clinics and hospital, 11%-18% had confirmed malaria. These estimates were based on light microscopy analysis of blood smears, the gold standard in malaria diagnosis in Thailand. However, microscopy is known for being insensitive at low-level parasitaemia [12], a scenario more and more common in areas of low and unstable transmission and in areas with declining trend for malaria [4].

In light of this, and of reports on high prevalence of subpatent asymptomatic infections in other regions [13-19], the objective of the present study was to obtain a more accurate assessment of the current epidemiology of falciparum and vivax malaria in western Thailand, where the country is setting the goal of malaria elimination by 2030. It is generally known that as malaria transmission declines, an increasing proportion of individuals are found to have asymptomatic and submicroscopic malaria infections. However, it is unknown the exact magnitude of prevalence difference detected by classic microscopic and the more sensitive PCR or qPCR methods, or serological markers. This is important because asymptomatic and submicroscopic malaria infections are known to contribute to transmission [20]. To begin elucidating this problem, in this preliminary study whole blood samples were collected from residents of a sentinel village and from patients at a malaria clinic in Tak province; they were screened for malaria parasites by quantitative PCR (qPCR) and plasma was probed on a protein microarray to detect plasma antibodies to over one-thousand *P. falciparum* and *P. vivax* proteins.

## Methods

### Study sites

The study was conducted in the northwestern Province of Tak in Thailand, on the bank of Moei River, bordering with Myanmar. The study sites are located 51 km apart: community samples were collected in the hamlet Mae Salid Noi (17° 28' 4.7202", 98° 1' 48.5106"), and malaria clinic samples were collected in the town of Mae Tan (17° 13' 49.0146", 98° 13' 55.6212"). The climate in this region is tropical. Average temperature ranges from 20.2°C in December to 29.3°C in April [11]. Rainy season is from May to early October with annual rainfall of 2,300 mm. Malaria transmission is low, unstable, and peaks in May-August, coincident with the rainfall [8]. In May 2012, expert microscopy analysis of blood smears collected during a comprehensive mass blood survey of the population of Mae Salid Noi (*n =* 558) detected one (0.17%) positive infection with *P. falciparum*. The predominant malaria vectors are *Anopheles dirus*, *Anopheles maculatus* and *Anopheles minimus.* Five malaria species that cause human infection are found in Thailand, but *P. falciparum* and *P. vivax* are vastly predominant [1,2,11,21,22].

### Study participants and ethical statement

Firstly, active case detection (ACD) surveys were conducted in Mae Salid Noi, and resident’s health status history was recorded weekly during a five-month period, from March to July 2012. For the molecular evaluation of parasite prevalence study, whole blood samples were collected during a community mass blood survey (MBS) in May 2012 from individuals aged >10 years old (range 11 to 95), resulting in 219 samples. This represented 39.2% of the population of the hamlet, from a total of 558 residents. This number of samples enabled us to detect 6.5% margin of error, using alpha 0.05. Secondly, 61 whole blood samples were collected from individuals >15 years old presenting at the malaria clinic of Mae Tan in May 2012, where passive case detection (PCD) is routinely conducted. All sample donors gave written informed consent. The study was approved by the Institutional Review Boards of the Pennsylvania State University protocol (number 34319); University of California Irvine (number 2012–9123); Thai Ministry of Health (number 0435.3/857), and EC of the Department of Disease Control Ministry of Public Health, Thailand protocol number 7/54-479.

### Sample classification

Samples were classified into four major categories, according to presence or absence of *Plasmodium* DNA by qPCR, and presence or absence of symptoms at the time of blood collection. Samples with qPCR result positive for Plasmodium DNA were classified as from 1) asymptomatic malaria, if donors presented no symptoms; or 2) symptomatic malaria, if donor presented symptoms as described below. Samples with negative qPCR result were classified as from 3) healthy individuals, if no symptoms were present; or 4) non-malaria illness, if symptoms were present. Malaria symptomatology was defined as fever (>37.5°C), fatigue, myalgia, headache and nausea, occurring alone or in combination.

### Blood sample collection and preparation

From each study participant, approximately 300 μl of whole blood was collected from a finger prick into a Microvette CB300 capillary blood collector with Lithium-Heparin (Sarstedt, Newton, NC), centrifuged to separate cellular and plasma fractions, then immediately frozen at −80°C for shipment to University of California Irvine for analysis. Upon thawing, plasma was removed, aliquoted and stored at −80°C until use. Total genomic DNA was isolated from the pelleted cellular fraction using DNeasy Blood and Tissue kit (Qiagen, Valencia, CA), and further purified with Genomic DNA Clean and Concentrator (Zymo Research, Irvine, CA), according to manufacturer’s instructions. Purified genomic DNA samples were kept at −20°C until use.

### Sample analysis by microscopy and quantitative PCR (qPCR)

Field microscopy is performed by local trained staff who provides the first result, and positive cases were treated per national malaria treatment guidelines. Expert microscopy was performed later at Mahidol University by an expert microscopist, with over three decades of experience [23], who provided the final microscopy result. Thin and thick smears were prepared from each blood sample, stained with Giemsa solution, and examined for >200 leukocytes for a thick film and >200 microscopic fields with a 100× objective. Molecular detection of *P. falciparum* (Pf) and *P. vivax* (Pv) parasites in blood samples was performed by qPCR in the 219 MBS and 61 PCD samples using a SYBR Green detection method as described in Rougemont *et al*. [24]. Species-specific primers were designed to detect *P. falciparum* and *P. vivax* 18S rRNA gene: for *P. falciparum*, the forward primer sequence was 5′-AGTCATCTTTCGAGGTGACTTTTAGATTGCT-3′ and the reverse was 5′-GCCGCAAGCTCCACGCCTGGTGGTGC-3′; for *P. vivax*, the forward primer sequence was 5′-GAATTTTCTCTTCGGAGTTTATTCTTAGATTGC-3′ and the reverse was 5′GCCGCAAGCTCCACGCCTGGTGGTGC-3′. Amplification was performed in 20 μl reactions containing 2 μl of genomic DNA, 10 μl 2XSYBR Green qPCR Master Mix (Thermo Scientific, Waltham, MA), and 0.5 μM of each primer, in a CFX96 Touch Real-Time PCR Detection System (BIORAD, Hercules, CA). After initial denaturation at 95°C for 3 min, 45 cycles of 94°C for 30 sec, 55°C for 30 sec, and 68°C for 1 min were followed by a final step of 95°C for 10 sec. This was then followed by a melting curve from 65°C to 95°C with 0.5°C increments for 5 sec. Samples were tested in triplicates and a given sample was considered positive if the lower 95% confidence interval for adjusted Ct value was greater than 0. The detection limit of this method was 50 parasites/mL of blood.

### *Plasmodium falciparum* and *Plasmodium vivax* protein microarray

A protein microarray displaying 500 *P. falciparum* and 515 *P. vivax* polypeptides printed as *in vitro* transcription translation (IVTT) reactions was manufactured as described previously [25]. Gene accession numbers follow annotation published on PlasmoDB [26,27]. The protein targets on this array, named Pf/Pv500, were down-selected from larger microarray studies published [28-30] and unpublished data collected at UCI, based on seroreactivity and antigenicity to humans. Quality control of array slides revealed over 99% protein expression efficiency of *in vitro* reactions spotted, as determined by detection of N- and C- terminal polyhistidine and influenza haemagglutinin epitope tags, respectively. Protein amount was consistent between multiple subarrays, with signal intensity of anti-6xHis tag probing showing a minimal R^2^ = 0.78 and a maximal R^2^ = 0.93 between subarrays and slides. For large proteins printed on the microarray as overlapping polypeptides or individual exons, the exon position relative to the full molecule and the segment of the ORF are indicated when applicable [30]. Information on the microarray platform is publicly available on NCBI’s Gene Expression Omnibus and is accessible through GEO Platform accession number GPL18316. For *P. falciparum* polypeptides spotted on the microarray, the distribution of parasite life stage of maximum expression, based on information from PlasmoDB according to Le Roch *et al.* [31], is as follows: merozoite 24%, early ring 22%, late schizogony and early trophozoite 14%, early schizogony 11%, late trophozoite 8%, late ring 5% and unknown 2%. Life stage expression information is not available for *P. vivax* proteins at this time.

### Probing of plasma samples on the Pf/Pv500 microarray

From the 219 whole blood samples from the community MBS screened by qPCR for *Plasmodium* infection, a subset of samples were selected for Pf/Pv500 microarray probing, using the following criteria: i) being 15 years or older in age, and ii) had recorded lack of malaria symptoms (as defined above) during household visits two months prior and two months after blood collection. This yielded 93 samples. Sixty plasma samples from patients from the malaria clinic were also probed (one plasma of the original 61 clinic samples was compromised and not tested on the array). Twelve plasma samples from unexposed donors from the United States, with no travel history to malaria endemic regions, were used as controls for serology comparisons. Probing of plasma samples on the microarray was previously described in Baum *et al.* [32]. The raw and normalized data of antibody binding to proteins on the Pf/Pv500 microarray is publicly available through NCBI's Gene Expression Omnibus Series accession number GSE55265.

### Data analysis

For analysis of antibody binding to Pf or Pv polypeptides on the microarray the following steps were taken: (i) the mean background signal of antibody binding to 24 control spots of IVTT reaction without DNA template (*no DNA control* spots) were subtracted from each plasma’s raw values of antibody binding measured as the mean signal intensity of spots of printed polypeptides; negative or zero values after background subtraction were assigned a net value of 1; (ii) net values were log_2_-transformed for data normalization. Normalized data was used for statistical analyses and for figure representations of the data; (iii) to determine which polypeptides were considered seroreactive by plasma from the Thai study cohort, Significance Analysis for Microarrays (SAM) [33] was performed comparing the intensity of antibody binding to the proteins on the array between the exposed plasma from Thailand (*n =* 153) and unexposed controls from the USA (*n* = 12). The test was performed using MeV 4.8.1, with the following parameters: median and 90th percentile of False Discovery Rate, 0.15% and 0.92%, respectively; median and 90th percentile of Number of False Significant Genes, 0.66 and 4.19, respectively. This resulted in 458 polypeptides being considered significantly seroreactive in exposed Thai plasma and all further analyses considered only this set. (iv) Individual plasma samples were considered seropositive for a polypeptide if the sample’s signal intensity value was above the upper 99% confidence interval value of the unexposed control group. For analysis of intensity of response, (v) ANOVA testing with *post hoc* Tukey-Kramer (Tukey’s Honestly Significant Difference, HSD) was used for pairwise comparison of the mean signal intensity amongst the plasma groups using JMP9.0; significance tests were 2-sided and set at the 0.05 level for type I error. (vi) Z-scores of signal intensity were calculated as the number of standard deviations above or below the mean signal intensity of the unexposed group. (vii) ROC analysis was performed using ROCR package for R to obtain AUC (area under the curve) values and Mann–Whitney *U* test values (Benjamini-Hochberg-corrected) for each seroreactive protein, in comparisons of intensity of antibody binding between samples from asymptomatic malaria (MBS) (*n* = 13) and symptomatic malaria cases (PCD) (*n =* 26) to identify serological markers significantly associated with asymptomatic infections.

## Results

### Infection rates and comparison between qPCR and microscopy results

Molecular screening by qPCR of 219 blood samples from the community of Mae Salid Noi detected *Plasmodium* DNA in 25, revealing 11.4% (95% CI 7.3 - 15.5%) malaria infections amongst villagers (Table 1). Ninety-two percent of these infections were asymptomatic; 2 qPCR-positive individuals were febrile at sample collection. There were no other instances of symptom complaints from study participants within two weeks prior and after time of sampling. No infections positive by qPCR were detected by microscopy, thus 25 (100%) *Plasmodium*-infected samples had submicroscopic parasite levels (Table 2).

**Table 1:** **Results of qPCR screening of samples from the community and clinic surveys**

	***P. vivax***	***P. falciparum***	**Pf + Pv mix**	**Positive**	**Negative**
**Community MBS** ***n =*** **219**	17 (7.7%)	8 (3.65%)	0 (0.0%)	25 (11.4%)	194 (88.6%)
**Malaria Clinic PCD** ***n =*** **61**	13 (21.3%)	7 (11.5%)	7 (11.5%)	27 (44.3%)	34 (55.7%)

**Table 2:** **Comparison of screening results between microscopy and qPCR for samples collected from community MBS and malaria clinic PCD**

		**Microscopy**							
		**Community MBS (** ***n =*** **219)**				**Malaria clinic PCD (** ***n =*** **61)**			
		**Pf**	**Pv**	**Pf + Pv**	**Neg**	**Pf**	**Pv**	**Pf + Pv**	**Neg**
**qPCR**	Pf	0	0	0	8	5	0	0	2
	Pv	0	0	0	17	0	8	0	5
	Pf + Pv	0	0	0	0	2	5	0	0
	Neg	0	0	0	194	0	0	0	34

Of 61 samples collected from symptomatic patients at the malaria clinic PCD, 27 (44.3%) (CI 32.6 – 56%) *Plasmodium* infections were detected by qPCR (Table 1), whereas microscopy identified 20 (32.8%) (Table 2); 7 (25.9%) samples from symptomatic patients had submicroscopic parasite levels. Infections with mixed *Plasmodium* species were detected in seven qPCR-confirmed infections; all were erroneously classified as single-species infections by microscopy (Table 2), making for 25.9% cryptic mixed-species infections. There was good agreement between qPCR and microscopy in the detection of negative samples and *P. falciparum* infections (Fisher exact p = 1.0 and p = 0.46, respectively). However, microscopy was significantly less sensitive to detect *P. vivax* infections than qPCR (p = 0.03).

### Binding of plasma antibodies to *P. falciparum* and *P. vivax* proteins

A subset of plasma samples from the community cross-sectional MBS (*n =* 93) and the clinic PCD (*n =* 60) were probed on a protein microarray displaying *P. falciparum* and *P. vivax* polypeptides. Of the community samples, 80 belonged to the healthy group and 13 to asymptomatic malaria group (three *P. falciparum*+, 10 *P. vivax*+). Of the malaria clinic samples, 34 were from non-malaria illness cases and 26 were from symptomatic malaria cases (six *P. falciparum*+, 13 *P. vivax*+, 7 *P. falciparum*/*P. vivax* mixed infections). Infection status of samples probed was kept blind until data analysis of array results.

Of the targets present on the microarray, 281 *P. falciparum* and 177 *P. vivax* polypeptides were recognized by plasma antibodies from Thai exposed individuals when compared to unexposed USA controls. To these polypeptides, the log_2_ mean signal intensity (SI) of antibody binding by exposed Thai plasma was 8.65 (CI 8.61 – 8.69), while the mean SI produced by unexposed control plasma was 3.50 (CI 3.37–3.63) (Mann–Whitney U p < .0001). The names and gene IDs of the 458 seroreactive targets of antibody response are listed in Additional file 1.

**Figure 1:**
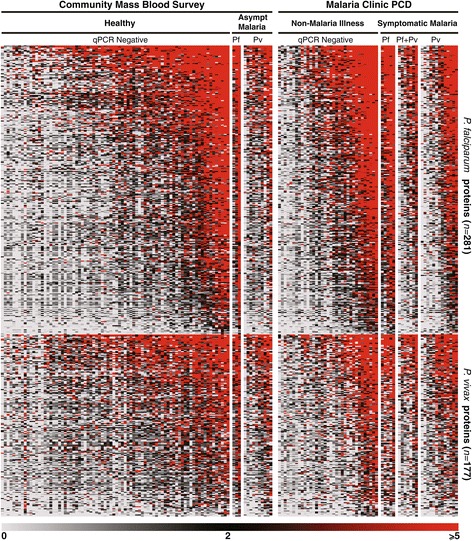
**Heatmap of signal intensity of antibody binding to seroreactive polypeptides on the microarray.** A three-colour gradient display of intensity of antibody binding to 281 *P. falciparum* and 177 *P. vivax* seroreactive polypeptides is shown for samples collected during a community-wide mass blood survey and passive case detection at a malaria clinic in Tak Province, Thailand. Samples are segregated according to infectious status and health condition (presenting symptoms or not) at the time of sample collection into four major groups: healthy, asymptomatic malaria, non-malaria illness and symptomatic malaria. Results from qPCR screening are shown as negative or the species (*Pf*, *Pv or Pf + Pv mixed-species)* identified in the sample. The coloured gradient represents Z-score values of signal intensity in relation to malaria-unexposed controls, ranging from 0 to ≥5. Individual samples appear as columns, ranked from left to right in their totals of binding to the array’s proteins; seroreactive polypeptides appear as rows, ranked from top to bottom in their mean values of antibody binding for all plasma.

Figure 1 shows the heatmap of Z-scores of signal intensity of plasma antibody binding to the seroreactive targets on the array. All Thai plasma samples probed (*n* = 153) were reactive to several polypeptides on the microarray, evidence of exposure to *Plasmodium* parasites in all sample donors. The median number of proteins recognized by a plasma sample was 297, and ranged from 54 to 457 antigens.

Seroprevalence rates for the seroreactive polypeptides are presented in Additional file 1, and ranged from 28% to 94%. Overall, the protein with highest seroprevalence was *P. vivax* MSP-10, which was recognized by 100% of qPCR-positive and >90% of qPCR-negative samples, from both MBS and PCD. The most frequently recognized *P. falciparum* proteins were ETRAMP-2, ETRAMP-5, heat shock protein 70, MSP-2 and MSP-4, Plasmodium exported protein PHISTc, ring exported protein 1 REX1, sexual stage specific protein precursor Pfs16 and conserved Plasmodium proteins PF3D7_1014100, PF3D7_0516400. For *P. vivax* proteins, the most frequently recognized antigens were ETRAMP, major blood stage surface protein Pv200, MSP-8 and MSP-10, sexual stage antigen s16 (putative), transmission blocking target antigen Pfs230 (putative), liver stage antigen (putative), endoplasmin precursor (putative) and hypothetical proteins PVX_092070, PVX_090110, and PVX_095185.

In pairwise multiple comparisons between plasma cohorts (asymptomatic malaria, symptomatic malaria, healthy and non-malaria illness) of their signal intensity of antibody binding to the 458 seroreactive proteins on the array, there was a homogenous response (HSD p > 0.05) to the majority of those targets (*n* = 380, 83%). The *P. falciparum* antigenic markers of exposure that were similarly recognized by all plasma cohorts, including plasma from qPCR-negative donors, were: apical membrane protein 1 (AMA1), several members of the early transcribed membrane protein family (ETRAMP 2, 5, 10.1, 10.2, 10.4), erythrocyte binding antigens 175 and 181, LSA1 and LSA3, several members of the MSP family (MSP 1, 2, 4, 5, 7, 10 and 11), heat-shock protein 70, duffy binding-like merozoite surface protein (DBLMSP), chromosome assembly factor 1 (CAF1), and several hypothetical Plasmodium proteins without known function, amongst others (Additional file 2). Of *P. vivax,* circumsporozoite protein, chitinase, several DNA repair proteins, putative dynein heavy chain, MSP 3alpha, 4, 7, 8 and 10, serine repeat antigens (SERA) 3, 4 and 5, amongst others, were equally seroreactive amongst plasma cohorts (Additional file 2).

### Comparison of antibody responses between community and clinic samples

**Figure 2:**
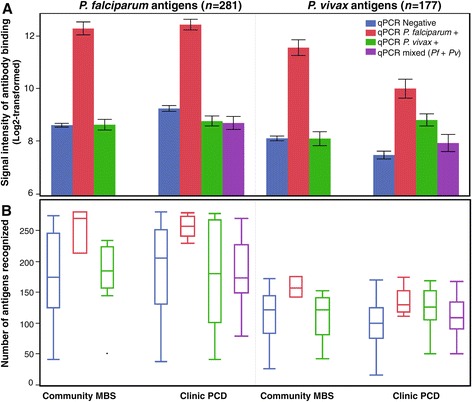
**Analysis of intensity and breadth of antibody response to** ***P.*** **f** ***alciparum*** **and** ***P. vivax*** **.** Plasma samples from the community MBS or malaria clinic PCD are segregated by qPCR result. **(A)** Average signal intensity of antibody binding to seroreactive polypeptides, shown as the mean log2-transformed signal intensity with 95% CI (error bars) for each plasma group. **(B)** Breadth of antibody response by each plasma group, shown as a box-whisker plot of the number of antigens recognized by plasma antibodies. Each box indicates the first and third quartiles, and the line inside the box is the median. The 1.5× interquartile range is indicated by the vertical line bisecting the box.

A bar chart of the mean signal intensity of antibody binding to the seroreactive falciparum and vivax proteins on the array is shown in Figure 2A. When samples from confirmed infections from both community MBS and clinic PCD were analysed, there were contrasting profiles in the magnitude of antibody binding observed according to the species of infecting plasmodia*.*

For *P. falciparum*-positive individuals (red bars), the intensity of antibody response to falciparum antigens was identical between asymptomatic (MBS) and symptomatic (PCD) cases (HSD p = 0.99); however, samples from asymptomatic carriers had significantly higher levels of antibody binding to vivax proteins than the clinic patients (HSD p = 0.001).

For individuals infected with *P. vivax* (green bars)*,* antibody binding to falciparum proteins was identical between cohorts (HSD p = 0.94); however, to vivax proteins there was significantly higher antibody response in symptomatic (PCD) cases than in asymptomatic (MBS) carriers (HSD p < .001).

Unexpectedly, non-infected individuals (blue bars) showed equal or higher antibody responses than those with *P. vivax* (green bars) or mixed-species (purple bars) infections in most comparisons (Figure 2A). Mixed-species infections were observed only amongst samples from PCD, and produced antibody responses that were significantly higher to falciparum antigens than to vivax.

The breadth of antibody response observed amongst the different plasma groups is shown in the box-whisker plot in Figure 2B. Falciparum proteins were more frequently bound by plasma antibodies than vivax proteins (Fisher exact p < .001), however there was no significant difference in the number of antigens recognized between the four plasma cohorts.

### Serological markers associated with asymptomatic infections

Receiver operating characteristic (ROC) analysis can be used to compare the sensitivity versus specificity of antibody binding to individual antigens for their ability to distinguish between two diagnostic groups. The antibody responses in asymptomatic and symptomatic malaria cases were compared using ROC analysis to determine which plasmodial proteins elicited higher antibody responses in asymptomatic infections and could be associated with protection from clinical disease in *Plasmodium* infections.

Table 3 presents the area under the curve (AUC) and Mann–Whitney U values for the protein spots that were significantly more reactive in asymptomatic infections than in those patients suffering disease symptoms. AUC values from 0.70 to 0.74 were obtained for six proteins. The highest AUC value was for *Pf* MSP2 protein, a well-known marker of clinical disease protection [34,35], followed by three conserved hypothetical proteins of *P. falciparum* and *P. vivax* of unknown functions. Finally, two *P. falciparum* proteins of putative functions, the chaperone of unfolded proteins or heat shock protein (DnaJ protein) [36], and the putative E1E2 ATPase, a cation-transporting P-ATPase [37] were also shown to elicit antibody responses stronger in asymptomatic carriers than in malaria patients.

**Table 3:** **Serological markers associated with asymptomatic infection**

**PlasmoDB ID**	**ORF fragment**	**Protein name**	**Organism**	**AUC**	**MW U**
PF3D7_0206800		merozoite surface protein 2 (MSP2)	*P. falciparum*	0.742	0.016
PF3D7_0703700	Exon 1 Segment 1	conserved Plasmodium protein, unknown function	*P. falciparum*	0.735	0.008
PVX_119695	Exon 3 of 3	hypothetical protein, conserved	*P. vivax* SaI1	0.725	0.013
PVX_085120	Exon 3 of 5 Segment 2	hypothetical protein, conserved	*P. vivax* SaI1	0.722	0.020
PF3D7_0806500	Exon 1 Segment 1	DnaJ protein, putative	*P. falciparum*	0.717	0.029
PF3D7_1348800	Exon 1 of 4	E1E2 ATPase, putative	*P. falciparum*	0.697	0.030

The intensity of antibody responses to these polypeptides was significantly higher in asymptomatic malaria cases when compared to symptomatic cases. AUC, area under the curve; MW U, Mann–Whitney U.

## Discussion

According to World Health Organization, Thailand is at the pre-elimination phase of malaria control [7]. The Government of Thailand has set a target to achieve >75% reduction in malaria case incidence by 2015 and 80% of the country areas to be free of locally acquired malaria transmission by 2020 [38]. To achieve such a goal, rapid detection and treatment of symptomatic infections, as well as identification of asymptomatic individuals and treatment of malaria reservoirs are paramount. These “silent carriers” are a challenge to malaria control efforts because they harbour the parasite and perpetuate transmission within the community, undetected. When malaria prevalence is low, rapid assessment of parasite exposure provides valuable information on transmission dynamics and whether the interventions being implemented are effective. Serology provides a view of the present as well as the recent past of parasite exposure, and seroprevalence rates can be used to define malaria endemicity [39,40] and distinguish between areas of differential exposure [32,41,42]. Protein microarrays, such as the one used in the present study, have been previously used to profile the antibody responses to hundreds of *P. falciparum* and *P. vivax* proteins simultaneously [28,30,32,43-47].

Thailand’s highest malaria burden regions, including the Tak Province where the present study was conducted, have experienced a drastic reduction in malaria transmission recently [11]. Parasite prevalence estimated by microscopy in 2011–2013 was below 1% in our study population, suggesting the area belonged to low-transmission region. If transmission was truly low, one would expect that the level of natural immunity in human population would be low, and symptomatic infections would be common. However, the profiling of antibody responses by microarray showed high seroprevalence, suggesting common exposure to malaria parasites, while qPCR detected 11.4% Plasmodium infection rate amongst villagers, 92% of which were asymptomatic. This was consistent with other studies on high prevalence of asymptomatic infections reported in the Amazon, Africa and Southeast Asia [13-19].

In our study, the sensitivity of microscopy was higher for blood smears from symptomatic individuals from the malaria clinic. However, microscopy misdiagnosed all *P.* falciparum/*P. vivax* mixed infections as single-species – mixed-species infections represented almost 26% of confirmed infections in patients at the malaria clinic. The underestimation of mixed-species infections in malaria patients by microscopy was previously documented [16,48-51], and failure to detect mixed-infections would result in inadequate or incorrect treatment, and may negatively affect the determination of malaria burden caused by falciparum and vivax malaria. The role of mixed-species infections in malaria transmission maintenance should be further examined [52,53]. Similarly, the importance of submicroscopic infections for malaria transmission is still unclear [54]. The Malaria Eradication Research Agenda suggested that any parasitaemia, no matter how small, may be potentially a source of transmission and thus a threat to malaria elimination efforts [4]. A meta-analysis performed by Okell *et al.* found that submicroscopic infections may contribute to 20-50% of transmission in areas where slide prevalence is 4% and below, but contribute considerably less in areas of high transmission intensity [20]. In many Southeast Asia countries and the Amazon, the majority of malaria infections are subpatent [13-16,19] as in the present study in Thailand. The detection limit of expert microscopy is generally 100 parasites per μL [12], whereas the high-sensitivity qPCR technique applied here detects as little as 0.05 parasites per μL of whole blood.

With the serological profiling using the protein microarray, the study’s objectives were two-fold: 1) to broadly describe the profiles of naturally acquired antibody responses of the study populations for the first time in such amplitude, correlating these findings with the epidemiology of malaria in the region; and, 2) to identify antigen-specific responses that could be serological correlates of protection from symptomatic manifestation during infection.

Firstly, the serological survey with a microarray showed surprisingly little differences in antibody responses amongst the four plasma groups tested, both in terms of antigen-specific responses and intensity or breadth of response. Seroreactivity to *P. falciparum* and *P. vivax* was detected in all the samples tested, including non-parasitemic individuals. Non-infected individuals exhibited overall similar levels of antibodies against plasmodia as individuals infected with *P. vivax* or mixed infections, indicating previous exposure to malaria parasites and possible maintenance of humoral immunity to infection. It is important to note that our cohort included only adults (range 15 to 95 years of age, median 33), and their serological profiles may reflect exposure to plasmodia from weeks to months past. Indeed, it is interesting that despite relatively higher prevalence of *P. vivax* in the region, antibody responses to *P. falciparum* were broader and more intense than to *P. vivax.* It is possible that the biology of falciparum infections may generate a stronger antibody response than vivax malaria, as seen in the *P. falciparum* + samples in this study (Figure 2A). It is also possible that this might be an effect from memory responses to *P. falciparum* in adult donors, which was relatively more prevalent in the region up to the mid-1990’s than *P. vivax* [10]. Perhaps these individuals have mounted a more robust antibody response to *P. falciparum* throughout those earlier years, which is maintained by “boosts” of occasional *P. falciparum* infections, or alternatively by *P. vivax* infections and re-activation of cross-reactive epitopes between the two species. The longevity of antibodies against plasmodia varies amongst antigens [55,56] and has both short- [57-59] and long-lived [60-62] components. Antibody cross-reactivity between P*. falciparum* and *P. vivax* was not addressed in this study due to the complexities of antigen-specific longevity of antibody responses, and the co-existence of these two species in the region, with the high likelihood of individuals having been infected with both at some point. Similarly, cross-reactivity between *P. ovale, P. malariae* and *P. falciparum, P. vivax* was not assessed.

Secondly, of the over 1,000 antigens analysed on the microarray, less than 8% of proteins elicited differential antibody responses amongst the plasma groups tested. Of those, only six showed significantly high responses associated with asymptomatic carriers and not with those who became sick. Once again, this likely resulted from studying solely the responses of adults, who have mounted a broad antibody repertoire throughout multiple exposures. Nonetheless, antibody responses to *P. falciparum* MSP2 showed the greatest ability to distinguish individuals with immunity to malaria disease from those who suffer symptoms when infected, confirming previous findings [34,35].

The main limitation of the serological study was the exclusion of plasma samples from children and adolescents. The inclusion of individuals younger than 15 years old in the serological survey would provide a better indicator of recent and current parasite prevalence, as they have not yet built a persistent antibody repertoire and possibly reflect more accurately the present picture of parasite exposure in the region. For the same reason, their serological profiles would possibly provide more distinguishing features for determining correlates of disease immunity [29]. Another general limitation of the study is the relatively small number of samples examined in the cross-sectional survey. The present work was not intended as a definitive study, rather as a preliminary assessment of the malaria epidemiology picture in the region according to molecular detection tools. A follow-up study in the same region is currently ongoing, which will examine by qPCR three seasonal samplings of the entire population of Mae Salid Noi.

Overall, the present findings suggest that low blood slide positivity rates in the community in Tak obtained by public health surveys should be interpreted cautiously in terms of malaria prevalence in the region, and that it may be imperative to include high-throughput molecular screening methods for malaria infection surveillance to identify infectious reservoirs or for evaluation of intervention program efficacy in the community. Although blood smear examinations by microscopy have lower cost for malaria detection, the use of sample pooling for PCR screening can bring the cost per sample down so that it can be considered for mass survey screening [63,64], with the advantage of gaining high sensitivity in detecting subpatent infections. Other methods, such as RFLP-dHPLC [16,65], multiplex qPCR [66] and LAMP [67] should also be considered. Additionally, for long-term monitoring of exposure as transmission levels drop further, serology may be a valuable tool, as detailed examination of age-specific seroprevalence profiles (seroconversion rates) can be used to monitor changes in transmission [40,68], and to detect transmission hot-spots [42]. Furthermore, absence of antibodies against Plasmodium has been used to show the success of elimination programmes in Mauritius [69], Greece [70], and in Vanuatu [71].

## Conclusions

Microscopic diagnosis grossly underestimates malaria exposure in Tak, Thailand. Our findings based on serological and qPCR surveys suggest that parasite prevalence is higher than currently estimated by local authorities based on microscopic screening of blood smears from community mass blood surveys or patients in clinics. As transmission levels drop in Thailand, it will be imperative to employ high-throughput methods with higher sensitivity for parasite detection in the phase of malaria elimination.

## Additional files

Additional file 1:
**List of seroreactive** ***P. falciparum*** **and** ***P. vivax*** **polypeptides recognized by plasma antibodies from exposed villagers and malaria clinic patients in Tak Province, Thailand, and their respective seroprevalence rates.**
Additional file 2:
**Pair-wise comparison of antibody binding intensity between plasma groups to seroreactive** ***P. falciparum*** **and** ***P. vivax*** **polypeptides on the microarray.**

## Abbreviations

MBS: Mass blood survey
ACD: Active case detection
PCD: Passive case detection
